# Resilience to Meet the Challenge of Addiction: Psychobiology and Clinical Considerations

**DOI:** 10.35946/arcr.v34.4.14

**Published:** 2012

**Authors:** Tanja N. Alim, William B. Lawson, Adriana Feder, Brian M. Iacoviello, Shireen Saxena, Christopher R. Bailey, Allison M. Greene, Alexander Neumeister

**Affiliations:** **Tanja N. Alim, M.D.,***is an assistant professor at the Department of Psychiatry and Behavioral Sciences, Howard University, Washington, DC.*; **William B. Lawson, M.D.,***is a professor and chair of the Department of Psychiatry at the Department of Psychiatry and Behavioral Sciences, Howard University, Washington, DC.*; **Adriana Feder, M.D.,***is an assistant professor at the Mood and Anxiety Disorders Program, Department of Psychiatry, Mount Sinai School of Medicine, New York, New York.*; **Brian M. Iacoviello, Ph.D.,***is a postdoctoral fellow at the Mood and Anxiety Disorders Program, Department of Psychiatry, Mount Sinai School of Medicine, New York, New York.*; **Shireen Saxena, M.S.,***Research associate at the Mood and Anxiety Disorders Program, Department of Psychiatry, Mount Sinai School of Medicine, New York, New York.*; **Christopher R. Bailey,***Research associate at the Mood and Anxiety Disorders Program, Department of Psychiatry, Mount Sinai School of Medicine, New York, New York.*; **Allison M. Greene, M.S.,***Research associate at the Mood and Anxiety Disorders Program, Department of Psychiatry, Mount Sinai School of Medicine, New York, New York.*; **Alexander Neumeister, M.D.,***is a professor in the Department of Psychiatry and Radiology, New York University Langone Medical Center, New York, New York.*

**Keywords:** Addiction, substance abuse, stress, acute stress reaction, chronic stress reaction, biological adaptation to stress, psychological response to stress, physiological response to stress, resilience, relapse, coping skills, psychobiology

## Abstract

Acute and chronic stress–related mechanisms play an important role in the development of addiction and its chronic, relapsing nature. Multisystem adaptations in brain, body, behavioral, and social function may contribute to a dysregulated physiological state that is maintained beyond the homeostatic range. In addition, chronic abuse of substances leads to an altered set point across multiple systems. Resilience can be defined as the absence of psychopathology despite exposure to high stress and reflects a person’s ability to cope successfully in the face of adversity, demonstrating adaptive psychological and physiological stress responses. The study of resilience can be approached by examining interindividual stress responsibility at multiple phenotypic levels, ranging from psychological differences in the way people cope with stress to differences in neurochemical or neural circuitry function. The ultimate goal of such research is the development of strategies and interventions to enhance resilience and coping in the face of stress and prevent the onset of addiction problems or relapse.

Evidence from different disciplines suggests that acute and chronic stress–related mechanisms play an important role in both the development and the chronic, relapsing nature of addiction (Baumeister 2003; Baumeister et al. 1994; Brady and Sinha 2005). Stress is defined as the physiological and psychological process resulting from a challenge to homeostasis by any real or perceived demand on the body (Lazarus and Fokman 1984; McEwen 2000; Selye 1976). Stress often induces multisystem adaptations that occur in the brain and body and affect behavioral and social function. The resulting dynamic condition is a dysregulated physiological state maintained beyond the homeostatic range. This definition and conceptualization of stress was further developed to explain the chronic abuse of substances and comfort foods and has been studied in the context of behavioral addiction (i.e., pathological gambling) (Dallman et al. 2005; Koob and Le Moal 1997; Koob 2003).

Persistent challenges to an organism through chronic substance use may ultimately lead to an altered set point across multiple systems. This hypothesis is consistent with evidence that suggests adaptations in brain reward and stress circuits, and local physiology (e.g., energy balance) can contribute to addictive processes. Cravings or urges, decreases in self-control, and a compulsive engagement in unhealthy behaviors each characterize patients with addiction (Dallman et al. 2005; Kalivas and Volkow 2005; Koob et al. 2004; Sinha 2001). Alternatively, a person’s ability to successfully cope with high stress is reflected in adaptive physiological and psychological responses (Charney 2004; MacQueen et al. 2003).

Resilience, defined as the absence of psychopathology despite exposure to high stress, can be studied by examining interindividual differences in stress responsivity across an organism’s various types (i.e., at multiple phenotypic levels). Responsivity ranges from psychological differences in the way individuals cope with stress to differences in neurochemical or neural circuitry function (Cicchetti and Blender 2006). Variability within the genetic makeup and quality of early-life experience, as well as interactions between the two, are known to contribute to differences in stress resilience (Enoch 2010; Heim and Nemeroff 2001). Genetic influences can stem from gene–environment interactions, changes in gene expression influenced by the environment (i.e., epigenetic changes), or variation within the actual genetic code. Some examples of genetic influences on resilience include variability in the genes involved in the body’s stress response (i.e., those controlling the hypothalamic–pituitary–adrenal [HPA] axis). These include those coding for the corticotropin-releasing factor (CRF) type 1 receptor or the glucocorticoid receptor (GR) (which cortisol can activate) as well as the serotonin transporter cathecol-O-methyltransferase (COMT), neuropeptide Y (NPY), and brain-derived neurotrophic factor (BDNF) genes (Feder et al. 2009) Genetic variation in the gene encoding the CRH1 receptor was found to moderate the impact of stress, for example, among adolescents engaging in heavy drinking (Blomeyer et al. 2008; Schmid et al. 2010). This gene-by-environment interaction predicted the initiation of drinking in adolescence as well as progression to heavy drinking by young adulthood (Schmid et al. 2010). The following sections highlight resilient responses to stress in studies in which stress was identified as an important factor contributing to the neurobiology of alcohol dependence.

## Psychosocial Factors Associated With Resilience

Early studies of children exposed to adversity (Masten 2001; Masten and Coatsworth 1998; Rutter 1985) as well as more recent studies in resilient adults (Ahmad et al. 2010; Alim et al. 2008; Bonanno 2004) have identified a range of psychosocial factors associated with successful adaptation to stressful or traumatic events. For example, the ability to simultaneously experience both positive and negative emotions when confronted with a high-stress situation increases flexibility of thinking and problem solving and can buffer individuals from developing stress-induced adverse consequences (Fredrickson 2001; Ong et al. 2006). Likewise, optimism has been associated with resilience to stress-related disorders, including alcohol use disorders (Ahmad et al. 2010; Alim et al. 2008).

Unlike personality characteristics associated with increased risk for substance use disorders (e.g., impulsivity, novelty seeking, and negative emotionality), positive emotionality, the tendency to experience positive mood frequently, was found to be associated with resilience to substance use in a large longitudinal study of public school students followed from late childhood through midadolescence (Wills et al. 2001). In this study, positive emotionality was found to buffer the effects of parent–child conflict and of parental and peer substance use on adolescent substance use. The ability to focus attention on performing and completing tasks was identified as a protective factor against substance use (Wills et al. 2001). The ability to focus attention might relate to the capacity to cope by planning and problem solving in times of stress, both types of coping styles characteristic of resilient individuals (Southwick et al. 2005).

Veenstra and colleagues (2007) examined the impact of coping style on alcohol use in response to stressful life events in a sample of 1,608 men and 1,645 women drawn randomly from the Dutch Lifestyle and Health Study (Veenstra et al 2007). Individuals who scored high on emotion coping, a coping style focused on feelings and emotional content to cope with stress, used more alcohol when experiencing a negative life event, compared with those who scored low on emotion coping. Alcohol use in times of stress did not vary by cognitive or by action coping, but the study found that cognitive coping and having more social contacts was linked to lower alcohol use in general. Another study of more than 1,300 adult drinkers in the general population from a New York county found stress-induced drinking in a subset of men (but not women) who scored high on avoidance coping and on positive expectancy from alcohol (Cooper et al. 1992). Men with low-avoidance coping and low expectancy from alcohol, on the other hand, actually showed a negative relationship between stressful life events and alcohol use. Of note, low avoidance coping has been linked to stress resilience in general, in several other studies (Alim et al. 2008; Carver et al. 1997).

## Neurochemistry of Resilience

**Figure 1:**
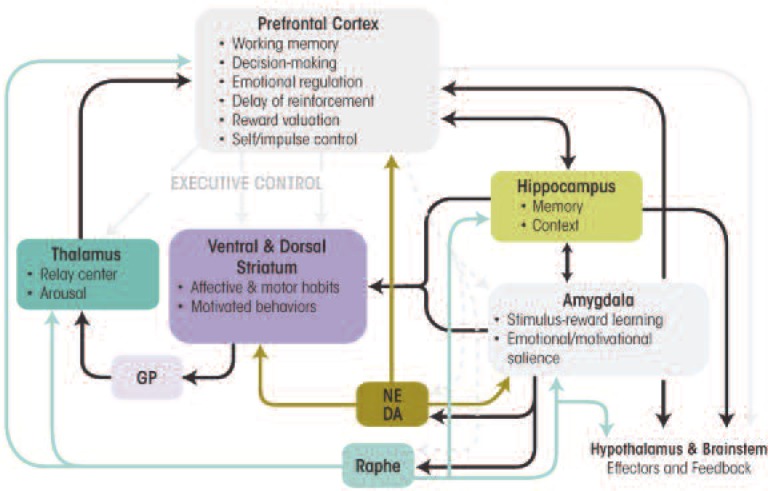
Norepinephrine (NE) and dopamine (DA) are the principle chemical messengers employed in central and peripheral sympathetic synapses, and the human NE transporter rapidly clears NE and DA from the synaptic cleft via efficient transport system-attenuating signaling, recycling 90 percent of these synaptic monoamines. NE neurons innervate nearly all parts of the neuroaxis, with the locus coeruleus (LC) being responsible for most of the NE in the brain. NE exerts neuromodulatory effects on the cellular activity of post-synaptic target neurons in many brain circuits, thereby moderating synaptic transmission in target circuits including the thalamus, prefrontal-cortex (PFC), ventral striatum (via PFC), and amygdala, which have been implicated in substance use disorders. The widespread and divergent anatomical organization positions the NE system to be involved in widely varying functions including responses to stress, which alters both the electrophysiological activity of NE neurons in the LC and the release of NE in the terminal regions of these cells, as well as crucial cognitive functions, including attention and arousal. NE mediates many of the adaptive and maladaptive consequences of stress exposure, implicating this system in a variety of abnormal behaviors including alcohol dependence.

“Allostasis” refers to the dynamic process through which the body adapts to daily stressors and maintains homeostasis (Sterling and Eyer 1988). Sudden stressful events trigger the release of the “flight-or-fight” hormones (i.e., catecholamines) and other stress hormones in the brain, preparing the organism to cope with stress and avert harm. This process is mediated by a stress circuit (see figure 1), which is consistently implicated in stress-related disorders such as mood and anxiety disorders and addictive disorders. Interindividual variability in stress resilience results from differences in the coordinated stress response. This response comprises the function and interactions of numerous hormones, neurotransmitters, and neuropeptides, some of which are discussed below.

### HPA Axis

The HPA axis is a system regulated by a complex negative-feedback system. CRF, released by the hypothalamus in response to stress, triggers the release of adrenocorticotrophic hormone (ACTH) from the anterior pituitary gland. This process leads to the synthesis and release of cortisol by the adrenal cortex. Cortisol secretion acutely facilitates cognitive, metabolic, immunologic, and behavioral adaptations to stress. It also results, however, in “allostatic overload” when stress becomes chronic or overwhelming (McEwen 2003). Resilience is maintained when the stress response is both activated and terminated efficiently. The adaptive responses of the HPA axis are thought to involve an optimal balance of the cortisol-binding receptors GR and mineralocorticoid receptor (de Kloet et al. 2005, 2007).

Studies showing lower plasma levels of ACTH but not cortisol in men with a family history of alcoholism (Dai et al. 2007; Gianoulakis et al. 2005) suggest that HPA axis dysfunction might predate the onset of alcoholism. Long-term alcohol abuse is associated with increased extrahypothalamic CRF signaling and dampened HPA axis responsivity (Richardson et al. 2008). Increases in extrahypothalamic CRF contribute to negative emotional states during abstinence, increasing risk for relapse (Koob and Le Moal 2008). In a recent study, researchers asked alcoholics who had been abstinent for 1 month to imagine a relaxing situation of their choice while listening to a previously recorded audiotape of this situation. A greater cortisol-to-corticotropin ratio (i.e., higher adrenal sensitivity) during this relaxed state was found to predict a shorter time to alcohol relapse, thus suggesting that new treatments aimed a decreasing adrenal sensitivity could reduce relapse rates (Sinha et al. 2011).

### Norepinephrine

During the acute stress response, the hormone norepinephrine (NE) is released through direct projections from the brain site where NE is synthesized (i.e., locus coeruleus) and other brain stem nuclei (i.e., structures that act as transit points for brain signals) into the amygdala, hippocampus, nucleus accumbens, prefrontal cortex (PFC), and other brain areas mediating emotional responses. Several studies have linked abnormal regulation of brain NE systems to stress disorders (Krystal and Neumeister 2009; O’Donnell et al. 2004). As drug dependence develops, levels of the neurotransmitter dopamine decrease and the NE stress system in the brain is activated, contributing to “stress-like states” and increased vulnerability to stressors during periods of abstinence (Koob and Le Moal 2008). In combination with CRF, NE also might contribute to the consolidation of emotional memories associated with drug use in the amygdala (Koob et al. 2009).

Stress resilience may be enhanced through the regulation of NE system responsiveness, which is mediated through effects on the NE transporter on catecholamine receptors (i.e., α2 adrenoreceptors), as well as interactions between the NE and other neurobiologic systems, such as the dopamine and serotonin systems (Krystal and Neumeister 2009). For example, animal studies have shown that PFC NE nerve cell projections (i.e., axons) have a latent capacity to enhance synthesis and recovery of transmitter, which might underlie the capacity to adapt to stress (Miner et al. 2006). This mechanism deserves further study in humans with positron emission tomography (PET), which uses positron-emitting radiotracers to show where and how compounds act in the brain (Ding et al. 2005). Other targets include the α2a and α2c receptors, which have complementary roles in the regulation of stress responses (Small et al. 2000). Yohimbine, a drug that blocks the α2 receptors (i.e., a receptor antagonist), increases alcohol self-administration and induces reinstatement of alcohol seeking (Le et al. 2005; Marinelli et al. 2007). The recent finding that an α2c receptor polymorphism (Del322–325) reduces feedback inhibition of sympathetic NE release (Neumeister et al. 2005) as well as evidence from studies in mice bred to have an inactivated α2c receptor (i.e., knockout mice) (Sallinen et al. 1999), suggest that interventions targeting this receptor might modulate stress and anxiety responses.

### Serotonin

The serotonin (5-HT) system, which consists primarily of neurons from the dorsal raphe nuclei that project widely throughout the brain (including the amygdala, ventral striatum, and PFC), is involved in the regulation of stress and anxiety. Serotonin has an important role in promoting neuroplasticity in the central nervous system, both during development and in adulthood. Serotonin also regulates the neurochemical effects of drugs of abuse, including alcohol, and is involved in modulating impulsivity, known to increase risk for alcohol and drug abuse (Kirby et al. 2011). The 5-HT system is itself modulated by drugs of abuse. For example, alcohol administration elevates 5-HT levels in the nucleus accumbens, ventral tegmental area (VTA), amygdala, and hippocampus, an effect that is more pronounced in alcohol-preferring rats. Reduced activity of the 5-HT system might contribute to depression during withdrawal and increase vulnerability to relapse (Kirby et al. 2011). In studies of macaques, differential function of the 5-HT system in interaction with early life stress was found to affect alcohol consumption: peer-reared female macaques with a specific variant (i.e., the l/s genotype) of the serotonin transporter polymorphism showed higher levels of ethanol preference and increased consumption over time (Barr et al. 2004).

**Figure 2:**
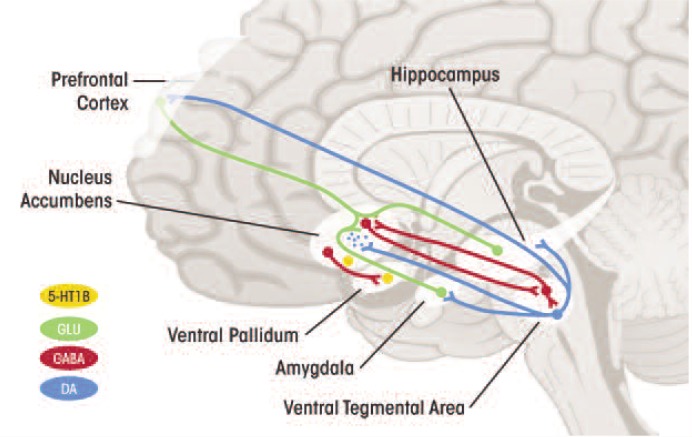
Alterations in serotonin 1B receptor (5HT_1B_R) function might contribute to alcohol dependence by influencing not only serotonin (5HT) input to the ventral striatum via the receptors’ role as 5HT terminal autoreceptors,^1^ but also dopaminergic input to the striatum via the role of these receptors as heteroreceptors^2^ on GABA terminals within the ventral tegmental area, and glutamatergic activity within the ventral striatum via heteroreceptors on corticofugal projections. ^1^ Autoreceptor: A site on a neuron that binds the neurotransmitter released by that neuron, which then regulates the neuron’s activity. ^2^ Heteroreceptor: A site on a neuron that binds a modulatory neuroregulator other than that released by the neuron.

The 5-HT system is extremely complex, including at least 14 receptor subtypes. Of these receptors, the 5-HT1_A,_ 5-HT1_B,_ 5-HT2_A,_ and 5-HT2_C_ receptors are well understood through research on anxiety regulation in both animals and humans (Krystal and Neumeister 2009). The 5-HT1_A_ receptor is thought to counteract the deleterious effects of 5-HT2_A_ receptor activation (i.e., the disruption of brain cell creation), mediated by increased release of the neurotransmitter glutamate and direct glucocorticoid effects (Hoebel et al 2007). Restrained function of another 5-HT receptor, 5HT1_B_, might be central to resilient stress responses by enhancing synaptic availability of 5-HT in the amygdala and other cortical regions as well as promoting dopamine release in the ventral striatum (Clark and Neumaier 2001; Krystal and Neumeister 2009; Sari 2004) (see figure 2).

The role of this receptor subtype in addiction disorders recently was studied in humans. The report demonstrated that alcohol dependence in humans, like in rodent models, is associated with increased levels of ventral striatal 5-HT_1B_ receptors (Hu et al. 2010). Additional research is necessary to understand the complex function of the 5-HT system. However, these findings suggest possible novel targets for the treatment of stress-related disorders and, most important, addiction disorders.

### Dopamine

Dopaminergic neurons in the ventral tegmental area (VTA) of the midbrain project to the nucleus accumbens and other limbic areas to form the mesolimbic dopamine system, the most studied reward circuit. Dopamine neurons are activated in response to reward or the expectation of reward, and generally are inhibited by aversive stimuli. Dopamine signaling is central to the onset of addiction, as well as to the transition to dependence in interaction with other neurotransmitter systems (Ross and Peselow 2009). Drugs of addiction trigger large but brief increases in extracellular dopamine in the nucleus accumbens. Over time, chronic drug use downregulates dopamine receptors and dopamine release, leading to decreased sensitivity to natural rewards, such as food and sex, and leading also to further drug use (Volkow et al 2010).

Although findings from animal studies suggest that early-life stress can lead to long-lasting changes in gene expression in the mesolimbic dopamine pathway, ultimately increasing vulnerability to addictive disorders, not all individuals with a history of childhood abuse develop addictive or other disorders, thereby stressing the role of protective factors such as genetic variants conferring resilience (Enoch 2010).

Findings from several studies suggest that higher dopamine D2 receptor availability in the striatum might promote resilience to alcohol use disorders. In a study of unaffected members of alcoholic families, higher striatal dopamine D2 receptor availability was associated with higher positive emotionality, discussed above as a protective factor against alcohol use disorders (Volkow et al 2006). Other studies found that higher striatal dopamine D2 receptor availability was associated with resistance to the reinforcing effects of stimulants in healthy volunteers (Volkow et al. 1999, 2002) and in rats (Thanos et al. 2008).

### NPY

NPY, a 36–amino acid peptide, is widely distributed in the brain. NPY has anxiety-reducing properties in rodents and is thought to enhance resilience to stress in humans (Feder et al. 2009; Morgan et al. 2000). Evidence from animal and human studies suggests that NPY has a key role in regulating alcohol intake, dependence, and withdrawal. Mice genetically modified to overexpress NPY consume less alcohol (Thiele et al. 1998), and administration of NPY into the cerebral ventricles of the brain (i.e., intracerebroventricular infusion) reduces alcohol consumption in alcohol-preferring rats (Thorsell 2007). Infusion of NPY into the central nucleus of the amygdala has been shown to normalize both anxiety behaviors and alcohol intake, suggesting that NPY might work by modulating anxiety responses (Zhang et al. 2010). In rhesus macaques exposed to early life stress, and in human studies, certain NPY gene polymorphisms are associated with differential susceptibility to alcohol or cocaine dependence (Koehnke et al. 2002; Lindell et al. 2010; Mottagui-Tabar et al. 2005; Wetherill et al. 2008).

### Endocannabinoids

An emerging body of evidence suggests an important role for the endogenous cannabinoid (eCB) system and specifically the CB_1_ receptor in alcohol-related behaviors (for review, see Basavarajappa 2007). To date, however, only peripheral measures of eCB function have been collected in living humans with alcohol dependence (AD) (Mangieri et al. 2009), and no human in vivo data on the potentially critical role of the brain CB_1_ receptor in AD have been collected yet. At a neurobiological level, studies show impairments in decision making in alcohol-dependent patients (Dom et al. 2006), which is associated with altered functions in a cortico-limbic-striatal circuit, including the amygdala, hippocampus, anterior cingulate cortex, insula, and the ventral striatum. Three sets of factors are thought to be responsible for high alcohol relapse rates. First, individual differences in the positive, reinforcing properties of alcohol are known to increase risk of alcoholism and possibly alcohol relapse (Schuckit and Smith 1996). Second, stimuli previously associated with alcohol use and its physiological and subjective effects become paired with alcohol and are thought to serve as “conditioned cues” that can increase alcohol craving and subsequent alcohol use (O’Brien et al. 1998). Finally, stress has been found to increase the risk of alcohol relapse (Brown et al. 1990; Miller et al. 1996; Sinha 2001). All three factors can be linked to the eCB system and its attending CB_1_ receptor and increasing evidence derived from animal studies suggests a role of the eCB system in alcohol-related behaviors (Vinod and Hungund 2006).

Such research suggests that upregulation of CB_1_ receptor–mediated G-protein signaling in a brain circuit that mediates AD susceptibility (involving the amygdala, hippocampus, ventromedial prefrontal cortex, insula, and ventral striatum) (Sullivan and Pfefferbaum 2005) might contribute to the increased alcohol consumption in patients with chronic AD. For example, CB_1_ inactivation (Hungund et al. 2003; Naassila et al. 2004; Poncelet et al. 2003; Thanos et al. 2005) and pharmacological manipulation of CB_1_ receptor function (Femenia et al. 2010; Maccioni et al.; Maccioni et al. 2008; Malinen and Hyytia 2008) result in reduced voluntary alcohol intake. In addition, administration of an agent that binds to the CB_1_ receptor (i.e., a CB_1_ receptor agonist) (Colombo et al. 2002; Gallate et al. 1999; Vinod et al. 2008*b*) enhances alcohol consumption.

In contrast, acute, short-term alcohol intoxication is associated with elevated eCB levels (Basavarajappa et al. 2006; Blednov et al. 2007; Vinod et al. 2008*a*), reduced activity of the enzyme fatty acid amide hydrolase (FAAH), and reduced CB_1_ receptor–mediated G-protein signaling (Vinod et al. 2011). This mediates the activation of the mesolimbic dopaminergic system (Cheer et al. 2007; Hungund et al. 2003), which has been extensively studied in alcohol dependence. Evidence suggests a functional interaction between these systems, which might be associated with the reinforcing effects of alcohol and therefore may be an important mechanism in the etiology of alcohol dependence. Findings in animal studies recently have stimulated interest in the therapeutic potential of enhancing eCB signaling, with research in humans having just begun (Hill et al. 2009). However, an accumulating body of evidence suggests that the eCB system, and in particular its attending CB_1_ receptor, provides novel leads for treatment development in alcohol dependence (Bailey and Neumeister 2011).

## Behavioral Interventions to Enhance Resilience

To date, most studies on resilience have been conducted in clinical populations with people exposed to traumatic life events as a prototype of stress-related disorders. However, these studies also can inform the development and implementation of behavioral interventions to address alcohol dependence. This is a critical application because the ultimate goal of research attempting to delineate a range of psychological, neurochemical, and brain circuitry mechanisms underlying resilience is the development of strategies and interventions aimed at enhancing resilience in the face of stress, which is of particular relevance for people struggling with alcohol dependence. As related to alcohol dependence, improving resilience would influence cognitive and emotional control in the face of stress, resulting in the ability to weather cravings without using alcohol, mindfulness to be aware of impulsive behavior and potentially avoid impulsive behaviors associated with alcohol use, and the development of prosocial behavior and interpersonal relations that could serve to support the individual in the face of stress and prevent alcohol use. Several cognitive and behavioral interventions have been developed in an effort to develop these capacities. These interventions, which include various forms of cognitive and behavioral psychotherapies (Butler et al. 2006; Marlatt 2001), mindfulness-based stress reduction (e.g., Astin 1997; Shapiro et al. 1998; Teasdale et al. 2000) and other therapeutic approaches, aim to help prevent the onset or minimize the extent of alcohol use behaviors. In addition, therapeutic approaches based on positive psychology might also help promote psychological resilience (e.g., Seligman and Csikszentmihalyi 2000) and are currently being evaluated for their effectiveness in addressing alcohol dependence.

Taken together, interventions aimed at enhancing resilience to stress that focus on developing cognitive reappraisal skills, fostering mindfulness, and facilitating social interaction that results in enhanced social support could be particularly effective in helping people cope with stress and preventing the onset of alcohol use problems or relapse. Indeed, cognitive–behavioral models of addiction and relapse treatment such as those provided by Marlatt and colleagues (e.g., Marlatt 2001) highlight the role of experiencing negative affect as a primary trigger for using alcohol and relapsing. Mindfulness skills can be particularly useful in helping an individual cope with negative affect in the moment without resorting to the use of substances. Moreover, the attributions that individuals make upon relapsing (whether the attribution for use is internal and stable: “I just can’t handle stress and I’m bound to keep using”—versus external and unstable: “This was really stressful and difficult to deal with, and I decided to take the easy route this time”) can influence whether the relapse develops into a full-blown relapse or remains an isolated event. Cognitive reappraisal of these situations and the attributions that individuals make of their alcohol use can thus be of great importance in developing resilience in the treatment of alcohol use disorders.

## Conclusions and Future Directions

Despite extensive research and knowledge regarding their serious adverse consequences, addiction disorders continue to contribute to the top preventable causes of death and morbidity in the United States (Centers for Disease Control and Prevention 2003). The mechanisms underlying the persistent and compulsive engagement in these behaviors remain poorly understood. Based on previous evidence, researchers have hypothesized that the chronic nature of addiction disorders is rooted in the neurotoxic effects of stress on the brain. These effects undermine the neuroplasticity within networks required for the recovery process to take place. As a result, mechanisms of resilience are crucial to the understanding of neuroadaptive potential and its behavioral consequences. This is an important topic of current research, which stands at a unique crossroad in the study of addiction disorders. The explosion in the field of molecular and cellular neuroscience calls for interdisciplinary, collaborative team-based approaches. A greater understanding of the neurobiology of stress and resilience, as well as its implications on the neurobiology of addictions, is crucial to the prevention of such disorders and to the development of evidence-based treatment strategies.
